# Supplementary material for: The bilirubin-to-albumin ratio as a potential prognostic biomarker for all-cause mortality in patients with acute decompensated cirrhosis: A prospective study
Source: PLoS One. 2025 Dec 22;20(12):e0337206. doi: 10.1371/journal.pone.0337206 (PMC12721523; doi:10.1371/journal.pone.0337206)
Supplement: S1 Fig — (PDF) [file pone.0337206.s001.pdf]

The bilirubin-to-albumin ratio: A potential biomarker for predicting all-cause mortality in acute decompensation patients with liver cirrhosis

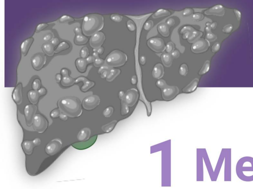

1 Method

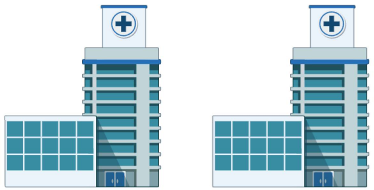

Two medical centers

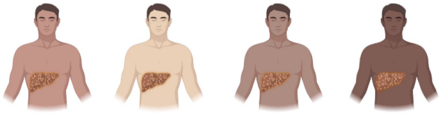

279 patients with acute decompensation

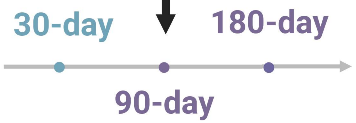

Follow-up for all-cause mortality

2 Results

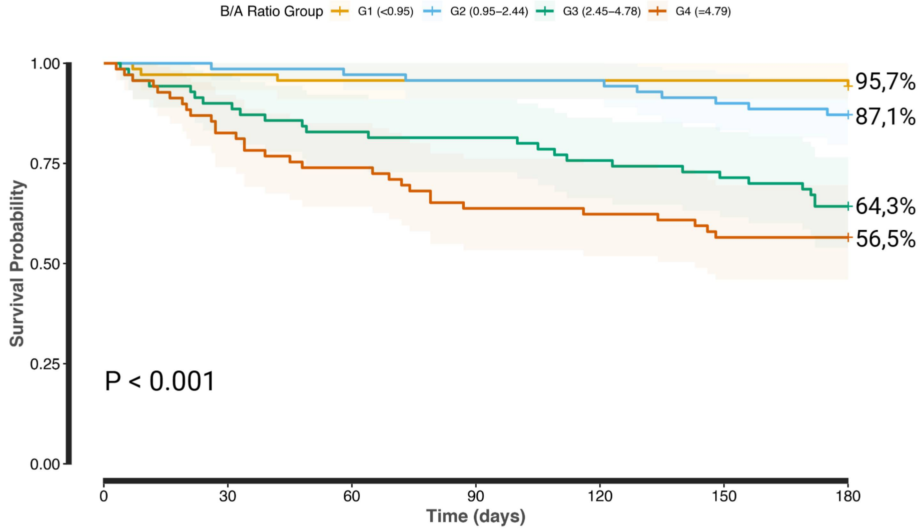

Number at risk

B/A Ratio Group	0	30	60	90	120	150	180
G1	70	68	67	67	67	67	67
G2	70	69	68	67	67	63	61
G3	70	63	58	57	53	50	45
G4	69	57	51	44	43	39	39

Time (days)

3 Conclusion

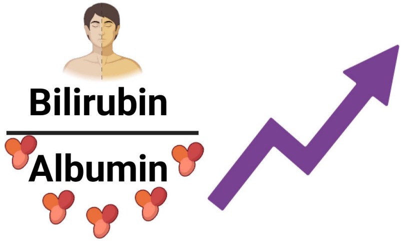

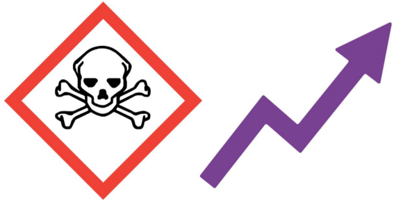

An elevated B/A ratio is linked to higher all-cause mortality in patients with acute decompensation of liver cirrhosis, suggesting its potential as a simple, accessible, and cost-effective prognostic marker.
