# Supplementary material for: The bilirubin-to-albumin ratio as a potential prognostic biomarker for all-cause mortality in patients with acute decompensated cirrhosis: A prospective study
Source: PLoS One. 2025 Dec 22;20(12):e0337206. doi: 10.1371/journal.pone.0337206 (PMC12721523; doi:10.1371/journal.pone.0337206)
Supplement: S1 Table — (DOCX) [file pone.0337206.s002.docx]

**Supplementary Table 1. Baseline clinical characteristics**

| **Variables** | **Total (n = 279)** | **ACLF* (n = 115)** | **AD (n = 164)** | **P value** |
| --- | --- | --- | --- | --- |
| Ages (years) | 55.00 ± 11.03 | 54.37 ± 11.87 | 55.43 ± 10.41 | 0.441 |
| Male (%) | 219 (78.49) | 90 (78.26) | 129 (78.66) | >0.99 |
| SBP (mmHg) | 120 (110 - 130) | 120 (110 - 130) | 120 (110 - 130) | 0.453 |
| DBP (mmHg) | 70 (70 - 80) | 70 (60 - 80) | 70 (70 - 80) | 0.717 |
| BMI (kg/m²) | 21.33 (19.53 - 23.74) | 22.06 (19.96 - 24.01) | 20.86 (19.15 - 23.03) | **0.023** |
| **Medical history** | | | | |
| Alcohol consumption (%) | 199 (71.33) | 79 (68.7) | 120 (73.17) | 0.423 |
| HBV (%) | 132 (47.31) | 59 (51.3) | 73 (44.51) | 0.275 |
| HCV (%) | 30 (10.75) | 14 (12.17) | 16 (9.76) | 0.559 |
| Liver cirrhosis (%) | 234 (83.87) | 91 (79.13) | 143 (87.2) | **0.044** |
| **Risk factors for hospitalization** | | | | |
| Infection (%) | 52 (18.6) | 33 (28.7) | 19 (11.6) | **<0.001** |
| Gastrointestinal bleeding (%) | 52 (18.6) | 20 (17.4) | 32 (19.5) | 0.755 |
| Acute viral hepatitis (%) | 101 (36.2) | 55 (47.8) | 46 (28.1) | **0.001** |
| Alcohol abuse (%) | 81 (29.0) | 43 (37.4) | 38 (23.2) | **0.011** |
| Hepatotoxic drugs (%) | 61 (21.9) | 43 (37.4) | 18 (11.0) | **< 0.001** |
| **Clinical symptoms** | | | | |
| Jaundice (%) | 215 (77.06) | 115 (100) | 100 (60.98) | **< 0.001** |
| Subcutaneousbleeding (%) | 29 (10.39) | 18 (15.65) | 11 (6.71) | **0.027** |
| Nevusaraneus (%) | 71 (25.45) | 34 (29.57) | 37 (22.56) | 0.210 |
| Erythema nodosum (%) | 78 (27.96) | 29 (25.22) | 49 (29.88) | 0.419 |
| Hippocrate (%) | 42 (15.05) | 18 (15.65) | 24 (14.63) | 0.866 |
| Enema (%) | 108 (38.71) | 52 (45.22) | 56 (34.15) | 0.080 |
| Collateral circulation (%) | 48 (17.2) | 29 (25.22) | 19 (11.59) | **0.004** |
| Hyper liver (%) | 111 (39.78) | 57 (49.57) | 54 (32.93) | **0.006** |
| Dyspepsia (%) | 85 (30.47) | 46 (40) | 39 (23.78) | **0.005** |
| Gastrointestinal bleeding (%) | 95 (34.05) | 34 (29.57) | 61 (37.2) | 0.201 |
| Adominal pain (%) | 106 (37.99) | 51 (44.35) | 55 (33.54) | 0.080 |
| Hypersplenism (%) | 98 (35.13) | 33 (28.7) | 65 (39.63) | 0.074 |
| Ascites (%) | 238 (85.3) | 113 (98.26) | 125 (76.22) | **<0.001** |
| Hepatic encephalopathy (%) | 67 (24.01) | 42 (36.52) | 25 (15.24) | **< 0.001** |
| **Prognostic scoring systems** | | | | |
| B/A ratio | 2.45 [0.95 - 4.79] | 5.27 [3.73 - 10.37] | 1.08 [0.61 - 1.82] | **< 0.001** |
| ALBI | -1.09 [-1.74 - -0.69] | -0.69 [-1.00 - -0.42] | -1.52 [-1.97 - -1.05] | **< 0.001** |
| EZ-ALBI | -19.72 [-26.25 - -14.08] | -13.47 [-17.28 - -6.53] | -24.27 [-28.58 - -19.64] | **< 0.001** |
| PALBI | -4.02 [-4.49 - -3.79] | -4.37 [-4.75 - -4.05] | -3.76 [-3.88 - -3.57] | **< 0.001** |
| Child-Pugh | 10 [8 - 11] | 11 [10 - 12] | 8 [7 - 10] | **< 0.001** |
| MELD | 17.73 [11.91 - 22.67] | 23.33 [20.09 - 27.38] | 12.48 [9.87 - 16.03] | **< 0.001** |
| MELD-Na | 19.58 [12.05 - 25.54] | 25.97 [22.53 - 30.69] | 13.49 [8.74 - 18.86] | **< 0.001** |
| MELD 3.0 | 20.26 [13.65 - 25.77] | 27.09 [23.40 - 30.17] | 14.64 [12.18 - 19.58] | **< 0.001** |

The values are presented as the means ± standard deviations or numbers (%) as appropriate. Bold values indicate p ≤ 0.050. Abbreviations: ALBI, albumin-bilirubin grade; B/A ratio, bilirubin-to-albumin ratio; BMI, body mass index; EZ-ALBI, easy albumin-bilirubin grade; DBP, diastolic blood pressure; HBV, hepatitis B virus; HCV, hepatitis C virus; MELD, model for end-stage liver disease; MELD-Na, model for end-stage liver disease sodium; MELD 3.0, model for end-stage liver disease 3.0; SBP, systolic blood pressure; PALBI, platelet-albumin-bilirubin grade.

**ACLF criteria on the basis of the APASL diagnostic guidelines for ACLF*[1]*.*

1. APASL ACLF Research Consortium (AARC) for APASL ACLF working Party., Sarin SK, Choudhury A, Sharma MK, Maiwall R, Al Mahtab M, et al. Acute-on-chronic liver failure: consensus recommendations of the Asian Pacific association for the study of the liver (APASL): an update. Hepatol Int. 2019;13: 353–390. doi:10.1007/s12072-019-09946-3
