# Supplementary material for: The bilirubin-to-albumin ratio as a potential prognostic biomarker for all-cause mortality in patients with acute decompensated cirrhosis: A prospective study
Source: PLoS One. 2025 Dec 22;20(12):e0337206. doi: 10.1371/journal.pone.0337206 (PMC12721523; doi:10.1371/journal.pone.0337206)
Supplement: Attachment — Submitted filename: Response to Reviewers.docx [file pone.0337206.s004.docx]

**REBUTTAL LETTER**

Dear Editor and Reviewers,

We would like to express our sincere gratitude to the reviewers and the editor for their thorough review and insightful comments on our manuscript. Your valuable feedback has significantly helped us improve the quality and clarity of our work. We greatly appreciate your time and effort in providing constructive suggestions, and we are committed to addressing all the points raised to ensure that our manuscript meets the highest standards.

We would now like to respond to the specific comments and suggestions provided by the reviewers as follows:

**RESPONSE TO EDITOR:**

*#1:* *AUC curves for MELD, MELD-Na and MELD 3.0 and Child-Pugh should be presented, as it seem that MELD 3.0 is almost superior to Bilirubin/Albumin, albeit your study was too small to identify a significant difference. Without a power calculation to demonstrate that sample size was large enough to discriminate a difference in performance you cannot claim equivalence.*

**Response**:

We have addressed this point by including additional data in Supplementary Table 2, where the AUC values of the MELD, MELD-Na, MELD 3.0, and Child-Pugh scores for predicting 30-day, 90-day, and 180-day mortality are presented alongside the B/A ratio. Initially, the MELD-based models and Child-Pugh score exhibited slightly higher AUCs than the B/A ratio at the 30-day mortality endpoint. However, this advantage diminished over time, and the predictive performance of the B/A ratio became comparable at later timepoints.

Although our study sample is comparable to those of previous research in this field, we acknowledge that it may still be underpowered to detect statistically significant differences in discrimination performance across models. As you correctly pointed out, the absence of a formal power calculation limits our ability to claim equivalence between the B/A ratio and established prognostic scores. We have now clarified this limitation explicitly in the Discussion section. Thank you again for highlighting this important consideration.

*#2: Explore if etiology of disease matter for survival*

**Response**:

We are thankful for your valuable recommendation. In our Cox regression Model II, we have already adjusted for etiologies and precipitating factors, including key contributors to acute decompensation, as part of the fully adjusted model. To enhance clarity, we will elaborate on this aspect more explicitly in the Results section to ensure that readers fully understand the scope of adjustment.

We believe that conducting a separate analysis focusing solely on etiology-specific survival would be beyond the scope of our current study objectives and could potentially divert from the primary aim, which is to evaluate the prognostic utility of the B/A ratio in acute decompensation. Nevertheless, we appreciate your suggestion and consider it a valuable direction for future research.

*#3: Patient likely need to divide in ACLF and non-ACLF, and explore at least in an explorative analysis.*

**Response**:

We are thankful for your valuable recommendation. Initially, we did perform subgroup division between ACLF and non-ACLF patients (AD) and conducted comparative analyses. However, we initially decided not to include these data in detail to maintain a concise manuscript and avoid overburdening the reader with excessive content.

In light of your valuable suggestion, we have now reinstated these analyses in the Baseline Clinical Characteristics section and provided the detailed data in Supplementary Table 1. This approach ensures that readers who are specifically interested in the differences between ACLF and non-ACLF patients can access the relevant information, while preserving the overall clarity and focus of the main text. Thank you again for your helpful recommendation.

*#4: For patients with ACLF the Clif score should be given.*

**Response**:

We are thankful for your valuable recommendation. In response to your recommendation, we have included detailed information on relevant prognostic scores specifically for patients with ACLF, including CLIF-C ACLF, CLIF-SOFA, CLIF-C OF, Child-Pugh, MELD, MELD-Na, and MELD 3.0 in Supplementary Table 1. These additions provide a more comprehensive overview of disease severity and support further interpretation of outcomes in this subgroup. Thank you for highlighting this important point.

**RESPONSE TO REVIEWER:**

*#1: Please give a proper rationale how B/A ratio will add to the already established prognostic scores.*

**Response**:

We are thankful for your recommendation. Trong nghiên cứu của chúng tôi It is unsurprising that the bilirubin-to-albumin (B/A) ratio initially appeared independently associated with outcome after partial adjustment, yet lost statistical independence once all covariates were incorporated. Acute, critically ill populations are influenced by myriad interacting factors, and in real-world practice clinicians never rely on a single parameter to judge prognosis.

This rationale underpins the development of multi-component scores such as MELD, MELD-Na and MELD 3.0, which integrate several laboratory variables. The value of the B/A ratio lies in its simplicity: it offers a rapid, holistic snapshot while more complex calculations and additional tests are still pending. Our aim was therefore not to create a solitary marker that supplants existing tools, but to highlight a readily available index whose performance approaches that of more elaborate scores.

Clinical decision-making is analogous to diagnosis: no physician bases a diagnosis on one symptom or one test result. A meticulous examination is required, synthesising multiple clinical signs and investigations before reaching a final judgment. Likewise, we do not advocate replacing established clinical or laboratory assessments with the B/A ratio; rather, we propose it as a quick, initial risk-stratification aid in acute decompensated cirrhosis, prompting timely, decisive management while comprehensive scores are being calculated.

Accordingly, we deliberately use the qualifier “potential prognostic” in the title, and we now emphasise in the Discussion that the B/A ratio is intended to complement—not replace—other critical clinical and paraclinical information.

*#2: Please explain whether it is superior/inferior to the existing scores.*

**Response**:

As previously mentioned, our study did not aim to demonstrate the superiority of the B/A ratio over established prognostic models. We fully acknowledge that such a comparison would be inappropriate, as multi-component scores like MELD, MELD-Na, and MELD 3.0 incorporate a broader range of laboratory parameters and clinical information, which naturally enhances their prognostic accuracy.

Instead, our objective was to highlight the practical value of the B/A ratio—its simplicity, accessibility, and ease of use—as a rapid, integrative marker that can provide early prognostic insight while awaiting more comprehensive test results. While the B/A ratio may not outperform these established models, its comparable performance and clinical convenience make it a valuable adjunctive tool, particularly in resource-limited settings or when rapid decision-making is essential.

*#3: Please revise the discussion and expand, adding the similar studies and comparing it with yours.*

**Response**:

Initially, we intended to include comparisons with similar studies; however, the majority of existing literature on the bilirubin-to-albumin (B/A) ratio focuses on neonatal or pediatric populations. Due to fundamental physiological and clinical differences between children and adults, especially in liver disease progression and systemic responses, we believe that direct comparisons across age groups would be inappropriate and potentially misleading.

Therefore, rather than drawing comparisons with these pediatric-focused studies, we opted to emphasize the pathophysiological rationale behind the prognostic value of the B/A ratio in adults with acute decompensation. This approach, in our view, aligns more closely with the aims of our research and provides a more relevant context for interpreting our findings. Nonetheless, we fully agree that comparative discussion with future adult-focused studies will be essential as more evidence emerges.

In addition to the revisions outlined above, we have diligently addressed all reviewer and editor comments. We’ve also conducted a thorough review of the manuscript for grammatical precision, scientific terminology, reference accuracy, and unit consistency, adhering to journal guidelines. These efforts have significantly elevated the manuscript’s quality and clarity. We eagerly await your feedback on this revised version.

Thank you once again for your invaluable contributions.

Sincerely,

The Authors
