# Supplementary material for: The bilirubin-to-albumin ratio as a potential prognostic biomarker for all-cause mortality in patients with acute decompensated cirrhosis: A prospective study
Source: PLoS One. 2025 Dec 22;20(12):e0337206. doi: 10.1371/journal.pone.0337206 (PMC12721523; doi:10.1371/journal.pone.0337206)
Supplement: Attachment — Submitted filename: Response_to_Reviewers_auresp_2.docx [file pone.0337206.s005.docx]

**REBUTTAL LETTER**

Dear Editor and Reviewers,

We greatly appreciate your time and effort in providing constructive suggestions, and we are committed to addressing all the points raised to ensure that our manuscript meets the highest standards.

We would now like to respond to the specific comments and suggestions provided by the reviewers as follows:

**RESPONSE TO EDITOR AND REVIEWER:**

*#1:* *There are previous scores using Albumin and Bilirubin one including Platelets assessing the “liver reserve” such as ALBI, EZALBI, PALBI. While this is in the setting of HCC resection, especially ACLF is where liver reserve plays a role, and thus should be included in assessment. It would be important to demonstrate that a new score is either substantially simpler or substantially more accurate in predicting mortality. Of the above models, you can potentially skip the 1st one, as it does not include Albumin, but the others would need to be explore in comparison to the new score.*

**Response**:

We sincerely appreciate this insightful comment. In response, we have incorporated the suggested scores (ALBI, EZ-ALBI, and PALBI) into our analysis, as presented in Supplementary Table 3. The results show that the B/A ratio did not differ significantly from these established models in terms of predictive performance. This further supports the notion that the B/A ratio, despite its simplicity, achieves comparable prognostic accuracy to more complex scoring systems.

*#2: While the study may be too small, a power calculation, to indicate an ideal study size would still be required.*

**Response**:

In accordance with the suggestion, we have added a power calculation to the Methods section to indicate the ideal study size.

Thank you once again for your invaluable contributions.

Sincerely,

The Authors
