# Supplementary material for: The bilirubin-to-albumin ratio as a potential prognostic biomarker for all-cause mortality in patients with acute decompensated cirrhosis: A prospective study
Source: PLoS One. 2025 Dec 22;20(12):e0337206. doi: 10.1371/journal.pone.0337206 (PMC12721523; doi:10.1371/journal.pone.0337206)
Supplement: Attachment — Submitted filename: Response_to_Reviewers_auresp_3.docx [file pone.0337206.s006.docx]

**RESPONSE LETTER**

Dear Editor,

We greatly appreciate your time and effort in providing constructive suggestions, and we are committed to addressing all the points raised to ensure that our manuscript meets the journal’s standards.

We respond to each of your comments as follows:

**RESPONSE TO EDITOR:**

*#1:* *When exploring the role to Albumin to Bilirubin, you need to cite in the introduction the previously and more established model, do NOT Push them into a supplement. The key question for you paper is if your new score is relevantly differnt from established scores that were already subsequently externally validated.*

**Response**:

Thank you for this valuable suggestion. We have revised the introduction section to include citations of established models and previous. In addition, we have refined the introduction section to clearly define the aim of our study, explicitly stating that it compares this ratio with previously validated scoring systems.

*#2: You score does not need to be superior to previous score to make you paper acceptable, but it should be more clarly demonstarted how you score compared to other scores performs.*

**Response**:

We appreciate your clarification. Accordingly, we have expanded the discussion section to provide a clearer comparison between the bilirubin-to-albumin ratio and existing prognostic models.

*#3: Please also better explain what your saple size calculation is based on. I believe your study did not aim to assess prevalence of cirrhosis, but prediction of mortality related to bilirubin to albumin ratio.*

**Response**:

Thank you for this insightful comment. We have revised the method section to explain our sample size calculation. As there were no prior studies directly evaluating this ratio in our target population, we referenced recent related studies to establish a reasonable estimate for sample size determination.

Once again, we are deeply grateful for your constructive feedback and the opportunity to further refine our manuscript. Your comments have greatly contributed to strengthening the scientific rigor and presentation of our work. We sincerely hope that the revisions meet your expectations.

With our best regards,

The Authors
