# Supplementary material for: The bilirubin-to-albumin ratio as a potential prognostic biomarker for all-cause mortality in patients with acute decompensated cirrhosis: A prospective study
Source: PLoS One. 2025 Dec 22;20(12):e0337206. doi: 10.1371/journal.pone.0337206 (PMC12721523; doi:10.1371/journal.pone.0337206)
Supplement: Attachment — Submitted filename: Response_to_Reviewers_auresp_4.docx [file pone.0337206.s007.docx]

**RESPONSE LETTER**

Dear Editor,

We greatly appreciate your time and effort in providing constructive suggestions, and we are committed to addressing all the points raised to ensure that our manuscript meets the journal’s standards.

We respond to each of your comments as follows:

**RESPONSE TO EDITOR:**

*#1:* *Your supplemental table 2 & 3 needs to be part of the man manuscript, you should also include the information from supplemental table 2 & 3 into the abstract. For table 2 you may also want to show the p-value comparing your A/B score to MELD etc, as in supplemental table 3.*

**Response**:

Thank you very much for your comment. Initially, we were concerned that including too many tables and figures in the main text might increase the total page count. However, following your suggestion, we have incorporated the data from Supplemental Tables 2 and 3 into Table 3 of the main manuscript. In addition, the comparative AUC values between our index and established prognostic models have been added to Figure 6 for clearer visual presentation. We also updated the Abstract to include relevant information from these tables, while ensuring that the word limit requirements were not exceeded.

*#2: You may also want to point out, that you show that you demonstrate that ALBI score was not significantly better than your more simple score, that can be calculated by hand vs. ALBI score, most of us would need a calculator. Though you would admit that the EZ-ALBI score can be hand calculated. But you can point out also, that while it was developed for HCC, you show that is also works for de-compensated cirrhosis independent of HCC.*

**Response**:

Thank you very much for your comment. We have revised the Discussion section, specifically the part on clinical implications, to emphasize that previously developed indices can also be applied in this context based on the data from our study.

**JOURNAL REQUIREMENTS:**

1. If the reviewer comments include a recommendation to cite specific previously published works, please review and evaluate these publications to determine whether they are relevant and should be cited. There is no requirement to cite these works unless the editor has indicated otherwise.

2. Please review your reference list to ensure that it is complete and correct. If you have cited papers that have been retracted, please include the rationale for doing so in the manuscript text, or remove these references and replace them with relevant current references. Any changes to the reference list should be mentioned in the rebuttal letter that accompanies your revised manuscript. If you need to cite a retracted article, indicate the article’s retracted status in the References list and also include a citation and full reference for the retraction notice.

**Response**: We have carefully reviewed the references suggested by the reviewers and editors, and only cited those that are directly relevant to our study. Unrelated references were not included. In addition, we thoroughly rechecked our reference list to ensure accuracy and to confirm that none of the cited papers have been retracted. No retracted publications are cited in our revised manuscript.

Once again, we are deeply grateful for your constructive feedback and the opportunity to further refine our manuscript. Your comments have greatly contributed to strengthening the scientific rigor and presentation of our work. We sincerely hope that the revisions meet your expectations.

With our best regards,

The Authors
